# Usability and Acceptability of a Mobile App for Behavior Change and to Improve Immunization Coverage among Children in Pakistan: A Mixed-Methods Study

**DOI:** 10.3390/ijerph18189527

**Published:** 2021-09-09

**Authors:** Abdul Momin Kazi, Nazia Ahsan, Waliyah Mughis, Saima Jamal, Raheel Allana, Mehreen Raza, Sahrish Muneer, Muhammad Ayub Khan Mughal, Hussain Kaleemuddin, Fareeha Sameen, Rao Moueed Ahmed, Munir Abbasi, Lampros K. Stergioulas, Saad Ahmed Qazi

**Affiliations:** 1 Department of Paediatrics & Child Health, Aga Khan University, Karachi 74800, Pakistan; naziya.ahsan@aku.edu (N.A.); waliyah.mughis@aku.edu (W.M.); saima.jamal@aku.edu (S.J.); raheel.allana@aku.edu (R.A.); mehro_r2@hotmail.com (M.R.); sehrish.munir@aku.edu (S.M.); ayub.khan@aku.edu (M.A.K.M.); hussain.kalimuddin@aku.edu (H.K.); 2 Faculty of Electrical and Computer Engineering, NED University of Engineering and Technology, Karachi 75270, Pakistan; fareehasameen@gmail.com (F.S.); moueedrao@gmail.com (R.M.A.); saadqazi@neduet.edu.pk (S.A.Q.); 3 Surrey Business School, University of Surrey, Guildford GU2 7XH, UK; munirabbasi@yahoo.com (M.A.); lampros.stergioulas@gmail.com (L.K.S.); 4 Faculty of IT and Design, The Hague University of Applied Sciences, 2521 EN The Hague, The Netherlands; 5 Neurocomputation Lab, National Centre of Artificial Intelligence, Karachi 75270, Pakistan

**Keywords:** mHealth, routine immunization, artificial intelligence (AI), barriers, short message service (SMS), LMICs, Pakistan

## Abstract

Background: Pakistan’s immunization uptake rates are still significantly lower than anticipated despite several initiatives. Lack of awareness, forgetting about vaccination schedule, and vaccine misconception/misinformation are a few of the major drivers that mitigate the rates of immunization. The current COVID-19 pandemic emphasizes the importance of immunization. The significant reductions in regular childhood vaccination during pandemic have increased the risk of outbreaks of vaccine-preventable diseases. Concerns among parents over possibly exposing their children to COVID-19 during child visits may have contributed to the reported declines. Innovative and cost-effective mHealth interventions must be implemented in order to address the problem of inadequate immunization rates. In addition, it is also critical to understand the end user needs in order to reflect on the highly relevant essence of the customized healthcare experience. Objective: The aim of this study was to learn about caregivers’ attitudes toward the usability and acceptability of behavior-change smartphone applications (mobile phones) for improving immunization coverage in Pakistan. Methods: A mixed-method design was employed for this study. The study was conducted at Aga Khan University, Hospital. Parents visiting the Community Health Center for 6-week vaccination of their children were recruited. The study was conducted in two stages. Stage 1 consisted of qualitative interviews that grasped the parent’s attitudes and challenges to immunization, as well as their acceptability and accessibility of the smartphone-based behavior-change application to increase vaccine uptake. Stage 1 was followed by stage 2, in which data were collected through a questionnaire designed by using data from qualitative interviews. Results: The majority of participants agreed that immunization serves an important role in protecting their child from illnesses that cause morbidity and mortality. Almost all of them emphasized the importance of using a pre-appointment method at vaccination center in order to reduce the waiting time. Furthermore, participants were also interested in AI-based behavior modification applications related to immunization. They also wanted to have applications in their native language for better understanding and communication of related information. In our study, approximately 95.2 percent of participants agreed to accept SMS immunization updates, which was also reasonably high. Lastly, the majority of them identified forgetfulness as a significant contributor to regular immunization. Conclusion: To enhance the uptake of childhood vaccines, overall vaccination rates, and overcome barriers related to vaccination coverage, cost-effective and user-friendly mHealth AI-based smart phone applications are required to raise awareness regarding the continuation of vaccination service and the importance of timely vaccination. Parents’ experiences and attitudes must be considered while designing and evaluating the efficacy of mHealth-based interventions.

## 1. Introduction

Childhood routine immunization (CRI) is considered as an important achievement in public health that has drastically reduced morbidity and mortality globally [1]. Around 2–3 million deaths are prevented annually through immunization [2]. The unwavering incommensurate rates of morbidity and mortality owing to vaccine-preventable diseases (VPDs) among children < 5 years of age in developing countries urges the need for innovative and sustainable initiatives to resolve factors militating against achieving universal access to immunization [3]. Pakistan has one of the highest infant mortality rates of 69.3 per 1000 live births [4]. Around 50% of the deaths are due to VPDs in children < 5 years of age, comprising about 15% of the total population [5]. This issue has been exacerbated due to the COVID-19 pandemic [6]. Given the sheer number of infants born each year, improvement of routine immunization coverage is crucial [7]. Considering the potential of vaccines to save millions of children worldwide, Pakistan has not yet integrated this undertaking in its health system with the same degree of competence, coverage, and accessibility [8]. 

Though the Expanded Program on Immunization (EPI) has increased immunization coverage against vaccine-preventable diseases from 5% to 84% in Pakistan, around 58% of the children are still unimmunized and are at a greater risk of diseases [9]. The immunization program is facing multifold challenges such as program structure, logistics, weak capacity of human resources, and vaccine-related perspectives [10]. Community-level barriers include logistical challenges, family income, education, lack of awareness about and access to vaccines, as well as cultural, social, religious, and personal beliefs [11]. Some of the reasons for low immunization coverage are misinformation, transparency, and the trust of the public regarding vaccination safety [12]. Around 32.9% of vaccines were delayed due to prior reminders not being sent to the parents and 26.6% were due to caregiver forgetfulness [13]. The issues linked to inadequate vaccination coverage are more challenging to address and mostly require interventions. Pakistan is lagging relative to the internationally standardized immunization targets. The failure to accomplish these goals is troubling both from a global perspective and within Pakistan’s national healthcare environment [9].

The burgeoning field of mobile health (mHealth) has provided a potential solution to address immunization-related challenges in developing countries [14]. Amongst several domains of mHealth, a smartphone-based application offers a valuable platform for receiving vaccination communication, immunization schedules, outbreak alerts, reminders for overdue vaccinations, reporting of adverse events following immunization (AEFIs), and allows for the tracking of vaccination records [15].

The Coronavirus 2019 (COVID-19) pandemic has upended the health systems in Pakistan. The disruption of routine immunization services has caused a greater risk for secondary outbreaks of vaccine-preventable diseases and worsened immunization coverage [16]. Conversely, the pandemic has unfolded a promising future of digital health for improving Pakistan’s healthcare system due to the increasing mobile phone access and internet connectivity. The Pakistan Health and Demographic Survey (PDHS 2017–2018) has revealed about 94% of households’ access to at least one mobile phone with urban households having 97.5% and rural households having 91.6% of the access to mobile phones. There is a significant discrepancy in the percentage of women and men who own a mobile phone; 93% of men own a mobile phone, compared with just 39% of women [17]. Furthermore, according to a report, 28% of zero-income earners in Pakistan own a cell phone, with 58% living in urban areas, 57% being women, 85% having less than primary or no school, and 36% being between the ages of 15 and 25 [18]. Moreover, immunization coverage has been shown to improve through mobile phone-based interventions in Pakistan [19]. Nowadays AI is being used in the area of healthcare promoting health, wellbeing, and behavior change. However, there is a scarcity of data addressing the behavior-change initiatives and strategies that are effective at engaging participants and improving immunization coverage using a mobile application from Pakistan. There is a dire need to focus on research that examines the application of technologies and design rigorous approaches to understand the use of these mobile based interventions in revolutionizing the conventional health system. The acceptance and adoption of mobile technology are completely dependent on the end-user. Identification of the potential barriers influencing the acceptance of mobile app is crucial for developing and evaluating digital health interventions. Therefore, we aimed to understand the caregiver’s perspective regarding their usability and acceptability of behavior-change mobile applications (mobile phone) to improve immunization coverage in Pakistan. Moreover, this study could help support technology-driven healthcare innovations for improving immunization coverage and timeliness in developing countries as well. 

## 2. Methods

### 2.1. Study Design

A mixed-method study design was employed. The study was conducted between November 2019 and April 2020. The study was divided into two Phases. In Phase I, qualitative interviews were conducted in February 2020 to understand the perceptions and barriers of caregivers regarding immunization and their acceptability and usability related to the smartphone-based behavior-change application to enhance vaccination uptake. Data from qualitative interviews guided the designing of the questionnaire for the Phase II quantitative survey, which was conducted in March 2020. The study was carried out following the conceptual framework of a previously published study which generated customized mobile messages to boost immunization coverage [20].

### 2.2. Study Setting

The study was conducted at the Community Health Center (CHC) of a tertiary care hospital—the Aga Khan University Hospital in Karachi, Pakistan. The center provides a wide range of services, including vaccination, pharmacy services, family medicine clinics, health promotion, family planning, and women’s health services. The majority of the population availing these services belongs to middle and upper income groups. All study participants were recruited from the vaccination center, offering immunizations for infants, young children, and adults. 

### 2.3. Qualitative Component

#### 2.3.1. Eligibility Criteria

Caregivers of the newborns or infants visiting the vaccination center (either for 6, 10, or 14 week vaccinations according to the EPI schedule of Pakistan) were included in the qualitative phase. Caregivers were considered eligible for the study if (a) a parent or guardian or at least one person in the household had a working Android-based smart mobile phone, (b) could use an Android-based smartphone, and (c) were willing to participate in the study. 

#### 2.3.2. Qualitative Data Collection

To recruit the study participants, a convenience sampling technique was used, in which health care professionals at the point of triage directed the parents to study staff while they arrived for their child’s planned regular immunization appointments at the vaccination center. Qualitative data were collected through in-depth interviews (IDIs) with eligible caregivers. A semi-structured interview guide was developed in English and the local/national language (Urdu) and pre-tested before being applied for qualitative interviews. Interview questions were modified after each pilot interview through constant analysis of consecutive interviews. Data collection ended after 11 interviews when data saturation was achieved. All interviewees were briefed about the background and objectives of the study, followed by their consent to participate in the study voluntarily. Caregivers were asked open-ended questions related to their perception and attitudes regarding CRI, their preference for visiting tertiary care hospital, the perceived role of staff and services provided through the vaccination center, the usability and acceptability of a smartphone-based behavior-change application, the potential barriers to its usage, and their expectations from the application for CRI improvement. Each interview took about 30 to 45 min. All interviews were audio-recorded. One interviewer conducted interviews, while the other served as a note-keeper. 

#### 2.3.3. Qualitative Data Analysis

The data were transcribed verbatim from Urdu to English. The transcripts were read repeatedly and analyzed manually through Discourse analysis (DA) to interpret participants’ perception of the behavior-change application for CRI uptake and improvement. DA highlights the contextual meaning of language in conversation or culture. As technology in health is linked to social discourse, its use and value are embedded in social structures and processes, which are organized through institutions and practices. Hence, to understand the how and why of using the smartphone-based application to enhance CRI, discourse analysis was applied to the qualitative data. The transcripts were segregated into meaningful texts labeled as ‘Codes’ that were exhibited the study context. Codes were then examined and categorized to capture the manifest meanings to generate main themes. Subsequently, a detailed analysis of the main themes resulted in the emergence of sub-themes. Data analysis was carried out independently by two investigators and discussed finally to resolve discrepancies to reduce the researcher’s bias. In the final analysis, the focus was on understanding the underlying meaning of the transcripts, thus yielding an account of how mHealth-based behavior-change interventions could be integrated to improve CRI service uptake. The collected information paved the way for phase II quantitative data collection.

### 2.4. Quantitative Component

#### 2.4.1. Eligibility Criteria

The qualitative phase was followed by quantitative survey data collection. Another population of caregivers of the newborn or infants visiting the vaccination center at the Community Health Center (CHC) either for 6, 10, or 14-week vaccinations (according to the EPI schedule of Pakistan) were recruited. The study objectives were explained after obtaining the informed consent. One child per household was selected. In a household where there was more than one child (due for 10- and 14-week vaccination), a random selection was made.

#### 2.4.2. Quantitative Data Collection

A close-ended survey questionnaire was constructed in accordance with a previous study by Kazi et al. [21,22]. Based on that, the questionnaire was constructed. The relevance scale was subjected to analysis. Data were entered in SPSS 20.0 (IBM. Corp, Armonk, NY, USA). To check the reliability and consistency of the relevance scale created, Cronbach’s alpha coefficient was analyzed. Cronbach’s alpha scores: 0.955. Alpha analysis results were valid, >0.7. The quantitative survey was conducted with questions regarding (1) demographics and socioeconomic status, (2) child’s vaccination status, (3) caregiver’s mobile usage, (4) preferred language for SMS, and (5) barriers to routine immunization. Out of 128 caregivers approached, 105 consented to participate in the study. 

#### 2.4.3. Quantitative Data Analysis

The dataset was analyzed using SSPS Version 20 to derive frequencies and proportions of the categorical variables such as socio-demographic information, smartphone usage, and immunization.

### 2.5. Ethical Consideration and Data Confidentiality

The study protocol and associated study instruments, including consent forms in English and the local language, were approved by Aga Khan University’s Ethics Review Committee before the commencement of any study activities. The study was conducted following the Helsinki declaration and established guidelines. All participants were administered informed consent before participation. Participants were given the right to refuse to participate in the study or leave the study at any time; this did not affect any services provided at the health center. Data privacy and confidentiality was maintained at all times. The audio recording and transcripts had a unique identifier; original and backup files were archived in a password-protected server and were accessible to the study-specific personnel only. 

## 3. Results

### 3.1. Qualitative

**Figure 1:**
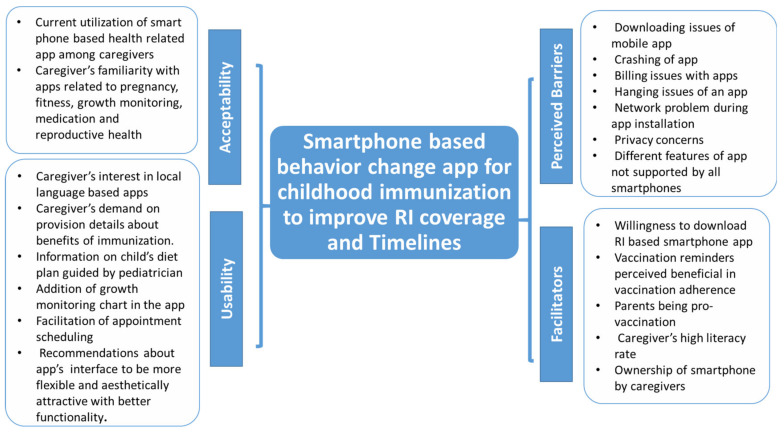
Perceptions of caregivers towards the smartphone-based behavior-change app for improving childhood routine immunization coverage.

Based on the qualitative data, the following main themes and subthemes emerged. Figure 1 encapsulates the overall enablers and barriers accentuated by the respondents for the utilization of the smartphone-based vaccination application. It further depicts caregivers’ current usability of health-associated mobile apps and their acceptability of the proposed behavior change app that could potentially increase immunization coverage rates and timeliness in our setting.

Table 1 presents the representative quotes for each theme that emerged from the qualitative data. While discussing the usability of the behavior change app, the majority of respondents believed that the app in the local language would be beneficial for those who are unacquainted with English. They perceived that health-related apps in the local/national language would help caregivers specifically to understand medical terms and can aid in their awareness of health-related issues. Others replied that the app should have both languages and their preferences should be optional for the end-users. Further to understand the barriers related to smartphone-based app, respondents were asked about the technical challenges they faced while downloading any app. Network issues during app installation were mainly highlighted by the caregivers. Concerns like the crashing of apps after installation, different types of features of the app not supported by all kinds of smartphones, and payment issues were also expressed. The hanging of mobile apps was another barrier identified by the mothers, along with server down complaints that resulted in irritation and discontentment by the app user. To understand privacy-related aspects of mobile apps, mothers were asked whether they review the terms and policies of any app before installing them on their mobile phone and if they ever read the health-based app’s privacy content for their child. The majority of respondents reported that they do not go through the policy-associated section of the applications, as they are at times too lengthy and do not contain any information worth reading. However, others acknowledged the importance of reading the privacy policy for security reasons as access to personal mobile data is a prerequisite in some apps. However, all the mothers were determined to read the privacy content of the vaccination app for the sake of their child’s health and considered it extremely important. Mothers were further asked about their basic knowledge and usage of any mobile phone-based app to gain an insight into their usage and acceptability of technology for health. Most of mothers self-reported to have previously heard about mobile-based health apps, while those remaining were completely unaware of it. Moreover, few mothers reported having ever downloaded any health app on their mobile phones. Those who downloaded them used them mainly during their pregnancy and found them helpful. One mother reported having used an in-built health app on her mobile phone that sent health-related information. Others stated to have downloaded mobile apps for monitoring blood pressure and menstrual cycles.

In addition to the views about the behavior-change app for RI, parents were also enquired about their preferences towards visiting the tertiary care hospital’s vaccination center, and perceived advantages and disadvantages for them compared to other facilities. Parental views highlighted their level of trust and satisfaction towards the quality of services and staff. Caregivers reported the health staff to be experienced, well trained, and cooperative. Alongside providing details of current vaccination, they were also guided about missed vaccines and following appointment schedule. The availability of vaccines at the facility was another facilitator highlighted by the caregivers. Because of these factors, parents preferred to visit CHC for vaccination in contrast to other facilities where services are free of charge. 

In contrast, long waiting and lack of sufficient space appeared to be the major concerns among mothers. Parents suggested employing a pre-appointment system for vaccination in contrast to the walk-in system to avoid long hours of waiting to receive the vaccination. Other caregivers complained that vaccination staff does not guide on the alleviation of the post-vaccine side effects at each visit; instead, this information is given at the first visit (i.e., at 6-week vaccination) only. To avoid any inconvenience and dissatisfaction at the client’s end, at every visit, counseling was recommended by the mothers.

Additionally, while sharing their views related to childhood routine immunization, the interviewees highlighted major areas, including the perceived benefits of immunization and immunization-related decision-making. Almost all the caregivers acknowledged the beneficial role of vaccination in a child’s health. They perceived vaccines as a preventive measure against various kinds of infectious diseases. Regarding immunization-related decision making, the majority answered that both parents decide about the health of their child. In a few cases, mothers were independently deciding, whereas in one case the father was the decision-maker for immunization-related issues in the family. The role of grandparents in children’s health was also highlighted, owing to the combined family system in Pakistan.

Finally, mothers expressed a great deal about the kind of expectations they have from the smartphone-based vaccination app. The majority of mothers demanded additional vaccine-related information from the app, such as notifications of vaccine availability at the vaccination center, doctors’ appointments for vaccination, vaccines to be administered after completing the EPI-recommended schedule, and introduction of any new vaccine for the children. Respondents perceived the vaccination app to be a valuable source of information for parents who are unaware of the advantages of immunization. Participants further acknowledged the significance of achieving the age-relevant milestone and expressed a lack of knowledge from other parents. Therefore, to keep the track of a child’s mental and physical growth, the idea of adding standard growth charts and age-appropriate milestones in the app was also put forward. The interviewee also expected the app to help parents find, consult, and receive a referral to professional healthcare providers who can be contacted in case of minor health-issues such as a blocked nose, congestion, cough, or colic issues. This could save parents from the hassle of visiting the clinics, saving time and resolving issues conveniently at home. Moreover, information on the danger signs for a child’s health and incorporation of interviews of pediatricians and experienced physicians were suggested to make caregivers more aware. Another mother recommended adding details about potential diseases of newborns and the children’s abnormal signs and symptoms. This could help parents detect early age syndromes not identified otherwise. This would also enable caregivers to recognize any issue with the child and seek medical help earlier. Some respondents appreciated the provision of religious content for immunization and perceived it essential for the behavior change of caregivers. Most mothers believed adding more pictures, colors, and videos that would make the vaccination app more likeable and interesting for caregivers. Besides, supplementation of relevant research articles on the child’s health and feeding issues would make it more convenient for parents to make them more confident and empowered about their child’s health. Respondents believed that new parents during the initial months are in a more information-seeking phase, so the app could be an attractive and helpful tool for them to take guidance from.

### 3.2. Baseline Survey

The sociodemographic characteristics of the participants are shown in Table 2. A total of 105 caregivers participated in the study. Among the caregivers, 53.3% (56/105) and 85.7% (90/105) of the primary caregivers were females. The monthly household income of 40% (42/105) of study participants was between 61,000–100,000 PKR. All of the study participants had access to a mobile phone; 99% (104/105) owned a smartphone. Among smartphone users, all of them, i.e., 100% (105/105) reported having the capacity to receive messages in a local language. Only 6.7% (7/105) reported network issues with their cellphone service provider. The preferred mode of communication of the majority of the participants was both SMS and phone call, i.e., 46.7% (49/105). Urdu was the most preferred language for the application, i.e., 81.9% (86/105). However, the education level of majority of the caregivers was post-graduation and graduation respectively, i.e., 59% (62/105) for fathers and 42.9% (45/105) for mothers. A prepaid network was used by 93.3% (98/105) of the caregivers. 

Table 3 summarizes participants’ experience and interest in receiving mobile phone reminders for routine immunization; 95.2% (100/105) of the participants preferred to receive SMS from the health center enquiring about their health and 78.1% (82/105) preferred a call from the health center as a facility. The expected frequency to receive messages from the clinic as a health facility was daily for 64.8% (68/105) of participants and the majority of them preferred the reminder-type of messages, i.e., 95.2% (100/105), to be received from the healthcare center. The majority, i.e., 98.1% (103/105) participants mentioned that currently they do not communicate with their healthcare provider through cell phone. The most common barrier that the caregivers were facing in getting their child immunized was forgetfulness 44.7% (47/105) and the majority of them did not face any difficulty in reaching the EPI CHC Vaccination Centre, i.e., 99% (104/105). 

## 4. Discussion

This study was based on semi-structured interviews followed by a baseline survey of the population visiting a vaccination center at a tertiary care hospital. The main objective was to explore caregivers’ perspectives and usability regarding an AI-based smartphone personalized application to improve immunization coverage. In LMICs, mHealth interventions are progressively being used for immunizations and other public health concerns. However, limited data are available regarding the adoption and engagement of caregivers with emerging health technologies. Understanding user capacity is necessary to incorporate new infrastructure into a conventional health system. This study is the first of its kind to understand caregivers’ perspective, usability, and acceptability of a behavior-change application for improving routine immunization coverage rates in resource-constrained settings through mixed methods. The qualitative interviews revealed several important themes based on potential benefits and barriers in immunizing children at tertiary care hospitals, parental belief, and decisions related to immunization, issues, and expectations associated with the mobile phone-based app. 

Parental decision-making regarding childhood vaccination is considered as an important scenario for health-related decision-making where parents have been stipulated to put major weight on the subjective perception of the outcome [23]. In this study, the majority of the participants responded that parents decide about the health of their child. They acknowledged the role of immunization in preventing children against diseases causing morbidity and mortality. Moreover, they shared that immunization is one of the most cost-effective interventions to curtail cost and burden on public health infrastructure.

Trust and rapport with a healthcare provider are pivotal for parental decision-making about vaccination [24]. This study’s qualitative data indicated that the majority of the participants reported trust and satisfaction regarding the vaccination services provided at the EPI center (CHC). Caregivers stated that the healthcare staff was experienced, well trained, cooperative, and provided adequate information regarding vaccinations. Most mothers indicated that their children’s safety and well-being were more important to them than the expense and for this reason, they opted for vaccination from a tertiary care hospital. The majority of the respondents highlighted the need to employ a pre-appointment system for vaccination at the center to avoid long waiting. Moreover, parental forgetfulness to make an appointment is an important factor that explains low vaccination coverage [25,26]. Since respondents from our study were already using smartphone devices, they emphasized the importance of such innovative tools in appointment scheduling and vaccine reminders. This could impact the overall quality of services provided by the vaccination center, making it more robust, convenient, and efficient. 

Almost all the caregivers were already familiar with mobile-based applications, including health apps. Few respondents reported having used the mobile apps for doctor’s appointments. Some of them used health apps for blood pressure monitoring, which they believed were not useful and stopped using. In addition, others noted that pregnancy apps, fitness apps, and apps for post-natal care were useful and valuable. The participants were also interested in the AI-based behavior-change application related to immunization. Most mothers showed an interest in apps that monitor their children’s emotional and physical development having user-friendly features. They also preferred to have applications in their native language to properly interpret the messages and related details. Overall, appointment scheduling, notification, vaccine reminder, useful information regarding the availability of the vaccine, and introduction of any new vaccine to a child’s schedule were found to be the major drivers for the utilization of the AI-based behavior-change application.

The quantitative baseline survey showed similar trends, i.e., around 85.9% of the total participants visiting tertiary care hospital had mobile phones. This was in accordance with the overall upsurge in cell phone usage in Pakistan. Rapidly increasing use of mobile applications have transformed many aspects of clinical practice including improvement in children’s health needs (e.g., vaccination reminders) [21]. The study results illustrated that caregivers were in favor of receiving both SMS and voice calls from the health center for their health-related concerns. This finding was in contrast with other findings, in which mothers preferred to receive voice reminders instead of SMS [27]. This could be related to the level of caregiver’s literacy and might serve as a barrier to SMS-based interventions. Most of the participants in our study were graduates from middle-income groups using smartphones, contrary to other study findings which showed high non-smart phone usage [28]. The willingness to receive SMS reminders for immunization in our study was 95.2%, which was also relatively high. All participants shared their mobile numbers with the study staff and were comfortable receiving SMS text messages in English and the local language, Urdu. In Nigeria, the study conducted depicted that the appointment reminders to mothers successfully enhanced vaccination coverage relative to the control group (RR = 1.72; 95% CI: 1.50–1.98) [29]. Most parents revealed forgetfulness as a major contributor to routine immunization non-adherence, rather than financial or socioeconomic factors. These results were similar to a Kenyan study’s findings that reflected the main reason of caregiver/guardians nonadherence to immunization schedule as failing to recall the immunization date [30]. 

Mobile phone and text message usage in Pakistan are quite high, with demographic data also showing at least one working mobile phone connection in households [22]. Technology cannot always be relied upon. Earlier research identified various barriers to the use of technology. These technological challenges could be a major barrier to both the usability and acceptability of the provided aid [31]. The potential barrier to app adoption identified in this study surrounded privacy issues, network issues during app installation, crashing of apps after installation, and unsupported app features in some smartphones. Moreover, all the participants in our study suggested that the vaccination app should have a privacy policy for security reasons and it should be easy to understand and should clearly indicate that the personal data is well protected. Overall, it was a mixed-method study based on the participant’s perception of how a behavior-change app should be. The results of this study will help the authors to create a behavior-change app with features in a local language that will improve routine immunization coverage in a low middle income county like Pakistan. 

The study has potential limitations. Because of the ongoing COVID-19 pandemic, the study design had to be modified to remote data collection, representing a pilot study with preliminary findings. Generalizability was another concern because this study was conducted in one immunization center of a private tertiary care hospital. Additionally, as immunization app usage was not tracked for the subset of mothers interviewed, this presents another limitation concerning the generalizability of their responses to the mobile phone-based immunization application. Another notable limitation is that the study was conducted in an urban area of Pakistan. The study population consisted of middle- and upper-income groups with no representation of low-income groups. Moreover, all the study participants were pro-vaccine individuals and more likely to download and use the app for immunization than those with less favorable attitudes. However, this does not negate the reported convenience of using a mobile phone-based application, providing the framework for further research on improving reminders for parents to complete CRI.

## 5. Conclusions

The exponential growth in smartphone utilization has also upsurged the advancement in mobile health applications. Mobile phones are proven to be helpful in addressing health issues within LMIC communities and bridging the gaps between accessibility to health information and the provision of services to large communities. While the majority of medical apps are prelude, surveys show that smartphone users have a clear preference for health apps that have created the ability to monitor and track information, access information wherever one is, and that appear authentic, useful, and reliable. The Android application can be helpful: phone-based reminders can be used by low-income, low digital literacy populations for improving vaccine uptake. Personalized reminders for parents are helpful in improving vaccine uptake. The findings of this assessment can be used to direct future studies on mHealth technologies to solve infrastructure, human capital, and behavior and technological issues before and after implementing the program. Qualitative evaluation of user perceptions should remain a priority in optimizing mobile data technologies to overcome regular immunization challenges in developing countries such as Pakistan and beyond. The study findings have potential to improve routine immunization in the health sector. It will ensure a time-saving, cost-effective approach to the productivity and delivery of safe, high-quality products.

## Figures and Tables

**Table 1:** Example of themes, quotes and recommendations from the qualitative study.

Theme	Quotes
I. Barriers and facilitators of the use of the CRI-based smartphone app (i) Perception of apps in the local language	“App should be in the local language as it could be useful for the majority of population who don’t understand English” (IDI 6) “Parents who don’t understand the medical terms in English can be benefitted from the health app in local language” (IDI 10)
(ii) Privacy concerns related to mobile apps	“I never read (privacy policy) because I don’t consider it important. However, I will read if it’s about my child’s health because I think differently about my child than myself” (IDI 4)
(iii) Downloading issues of mobile apps	“Yes I experienced issues in applications like when I have to pay for it, memory is-sues and sometimes applications gets crashed” (IDI 4)
II. Tertiary care hospital-based barriers for immunizing children	“They must counsel about side effects and post-vaccination care at every visit as other than the primary care-taker, someone else could bring the child for vaccination “(IDI 8)
III. Parental beliefs and immunization-related decision making	“Health is given high importance in our family. We always go for private vaccination as we believe not to spend much on what we eat but we always spend well on vaccination” (IDI 3) “Family elders (grandparents) takes the decision and guides us as well. For the first child, you don’t know much so they are the ones we seek guidance from” (IDI 2)
IV. Experience with health-related apps	“My mobile has one in-built health app which I have used once or twice. It gives notification related to weight and hypertension and other general information” (IDI 5)
V. Expectations from the smartphone-based vaccination app	The app should guide the purpose of immunization, make parents aware of its importance and the consequences on the child’s health if missed or delayed due to their carelessness” (IDI 11) “The app should be able to suggest whom to refer (doctor) and where to go when the child shows signs like dullness, not eating properly, gets tired, etc.” (IDI 1) “The app should be able to guide parents which milestone should be achieved at which age so they can easily monitor their children’s mental and physical growth” (IDI 11)
(i) Features of the EPI Reminder app	“One thing I do find interesting is religious information (on childhood vaccines) in the app as it seems missing from our current culture” (IDI 8) “This App should advise new mothers about taking care of their child if they are doing it single-handedly. Attractive pictures and video should be added to make it more engaging” (IDI 2)

**Table 2:** Socio demographic and mobile phone usability characteristics of the caregivers.

Variables	Frequency % (*n* = 105)
Gender	
Male	49 (46.7)
Female	56 (53.3)
Age	
Mother	Mean = 28; SD = 4.1 (Min = 19, Max = 44)
Father	Mean = 32; SD = 3.9 (Min = 25, Max = 48)
Qualification of child’s mother	
No formal education	1 (1.0)
Secondary/matric (9–10)	7 (6.7)
Intermediate (11–12)	9 (8.6)
Graduate (13–14)	45 (42.9)
Post-Graduate	43 (41.0)
Qualification of child’s father	
Middle (6–8)	1 (1.0)
Secondary/matric (9–10)	2 (1.9)
Intermediate (11–12)	4 (3.8)
Graduate (13–14)	36 (28.1)
Post-Graduate	62 (59.0)
Primary caregiver/guardian of child	
Father	12 (11.4)
Mother	90 (85.7)
Grandparent	3 (2.9)
Household Income (Rs.)/month	
Less than PKR 30,000	1 (1.0)
PKR 30,000–45,000	4 (3.8)
PKR > 45,000–60,000	15 (14.3)
PKR > 60,000–100,000	42 (40)
PKR > 100,000	28 (26.6)
Do not know	15 (14.3)
Ownership of the phone (participants)	104 (99.0)
Does your phone have the capacity to receive messages in local language text?	105 (100)
Type of Connection	
Prepaid	98 (93.3)
Postpaid	7 (6.7)
Preferred language for application	
Urdu	86 (81.9)
Preference mode of communication	
Texting	43 (41.0)
Talking	13 (12.4)
Both	49 (46.7)

**Table 3:** Participants’ acceptance in receiving mobile phone messages related to routine immunization.

Preference for SMS and Phone Call from Health Facility to Inquire about Their Health	
SMS	100/105 (95.2)
Call Preference for frequency of messages to be received from the clinic	82/105 (78.1)
Daily	68 (64.8)
Weekly	27 (25.7)
Monthly	10 (9.5)
Do you currently communicate with your health provider by phone?	
No	103 (98.1)
Preference for the type of messages	
Educational	3 (2.9)
Reminder	100 (95.2)
Adverse	2 (1.6)
Does insecurity prevent you from accessing the EPI center?	
No	104 (99.0)
Common possible barrier in getting the child immunized for routine immunization	
Forgot child’s due date for routine immunization	47 (44.7)

## Data Availability

Not applicable.

## Abbreviations

AI	artificial intelligence
CHC	Community Health Center
CoNTINuE	Capacity Building in Technology-Driven Innovation in Healthcare
EPI	Expanded Programme on Immunization
LMICs	low- and middle-income countries
PDHS	Pakistan Demographic and Health Survey
CRI	Childhood Routine Immunuzation
IDI	In depth interviews
DA	Discourse Analysis
